# Supplementary material for: Curated and harmonised transcriptomics datasets of interstitial lung diseases
Source: Data Brief. 2025 Oct 14;63:112139. doi: 10.1016/j.dib.2025.112139 (PMC12581653; doi:10.1016/j.dib.2025.112139)
Supplement: Supplementary file 1 [file mmc1.zip › Supplementary_material/RNA-seq/fastQC reports/GSE99621/SRR5642439_2_fastqc.html]

SRR5642439\_2.fastq.gz FastQC Report 

FastQC Report

Thu 1 Feb 2024  
SRR5642439\_2.fastq.gz

## Summary

- Basic Statistics
- Per base sequence quality
- Per tile sequence quality
- Per sequence quality scores
- Per base sequence content
- Per sequence GC content
- Per base N content
- Sequence Length Distribution
- Sequence Duplication Levels
- Overrepresented sequences
- Adapter Content

## Basic Statistics

| Measure | Value |
| --- | --- |
| Filename | SRR5642439\_2.fastq.gz |
| File type | Conventional base calls |
| Encoding | Sanger / Illumina 1.9 |
| Total Sequences | 20515588 |
| Sequences flagged as poor quality | 0 |
| Sequence length | 86-126 |
| %GC | 45 |

## Per base sequence quality

## Per tile sequence quality

## Per sequence quality scores

## Per base sequence content

## Per sequence GC content

## Per base N content

## Sequence Length Distribution

## Sequence Duplication Levels

## Overrepresented sequences

| Sequence | Count | Percentage | Possible Source |
| --- | --- | --- | --- |
| AGTACATGGGGAGGCATTGAGGCAGCCAGCGCAGGGGCTTCTGCTGAGGG | 57394 | 0.27975800644856 | No Hit |
| AAAAAAAAAAAAAAAAAAAAAAAAAAAAAAAAAAAAAAAAAAAAAAAAAA | 52374 | 0.2552888077105077 | No Hit |
| AGTACTTTTTTTTTTTTTTTTTTTTTTTTTTTTTTTTTTTTTTTTTTTTT | 39665 | 0.1933407904272595 | No Hit |
| AGTACATGGGCTGATGTTCGCCGACCGTTGACTATTCTCTACAAACCACA | 24534 | 0.11958711590425777 | No Hit |
| AGTACATGGGGAATAATTGCAATCCCCGATCCCCATCACGAATGGGGTTC | 23266 | 0.11340644976882944 | No Hit |
| AGTACATGGGGAGCGGTCGGCGTCCCCCAACTTCTTAGAGGGACAAGTGG | 21296 | 0.10380399528397626 | No Hit |

## Adapter Content

Produced by FastQC (version 0.11.7)
